# Device-Controlled Microcondensation for Spatially Confined On-Tissue Digests in MALDI Imaging of *N*-Glycans

**DOI:** 10.3390/ph15111356

**Published:** 2022-11-03

**Authors:** Annabelle Fülöp, Christian Marsching, Frederik Barka, Yasemin Ucal, Pauline Pfänder, Christiane A. Opitz, Günes Barka, Carsten Hopf

**Affiliations:** 1 Center for Mass Spectrometry and Optical Spectroscopy (CeMOS), Mannheim University of Applied Sciences, Paul-Wittsack-Str. 10, 68163 Mannheim, Germany; 2 SunChrom Wissenschaftliche Geräte GmbH, 61381 Friedrichsdorf, Germany; 3 Metabolic Crosstalk in Cancer, German Consortium of Translational Cancer Research (DKTK), German Cancer Research Center (DKFZ), 69120 Heidelberg, Germany; 4 Faculty of Bioscience, Heidelberg University, 69120 Heidelberg, Germany; 5 Neurology Clinic, National Center for Tumor Diseases, 69120 Heidelberg, Germany; 6 Medical Faculty, Heidelberg University, 69117 Heidelberg, Germany; 7 Mannheim Center for Translational Neuroscience (MCTN), 68167 Mannheim, Germany

**Keywords:** MALDI MSI, *N*-glycans, enzymatic digestion, micro-condensation, standardization

## Abstract

On-tissue enzymatic digestion is a prerequisite for MALDI mass spectrometry imaging (MSI) and spatialomic analysis of tissue proteins and their *N*-glycan conjugates. Despite the more widely accepted importance of *N*-glycans as diagnostic and prognostic biomarkers of many diseases and their potential as pharmacodynamic markers, the crucial sample preparation step, namely on-tissue digestion with enzymes like PNGaseF, is currently mainly carried out by specialized laboratories using home-built incubation arrangements, e.g., petri dishes placed in an incubator. Standardized spatially confined enzyme digests, however, require precise control and possible regulation of humidity and temperature, as high humidity increases the risk of analyte dislocation and low humidity compromises enzyme function. Here, a digestion device that controls humidity by cyclic ventilation and heating of the slide holder and the chamber lid was designed to enable controlled micro-condensation on the slide and to stabilize and monitor the digestion process. The device presented here may help with standardization in MSI. Using sagittal mouse brain sections and xenografted human U87 glioblastoma cells in CD1 nu/nu mouse brain, a device-controlled workflow for MALDI MSI of *N*-glycans was developed.

## 1. Introduction

Matrix-assisted Laser Desorption/Ionization Mass Spectrometry Imaging (MALDI-MSI) is a label-free technology for spatially resolved analysis of multiple classes of biomolecules in pharmaceutical and clinical research and the life sciences [1,2,3]. Whereas many small molecules such as metabolites, lipids, short peptides, or drugs can be conveniently analyzed using workflows that require few handling steps, others demand on-tissue chemical derivatization [4] or on-tissue enzyme digestion [5,6]. In particular, the spatially-resolved analysis of tryptic peptides [7,8] or *N*-glycans [9,10] from fresh-frozen or formalin-fixed paraffin-embedded (FFPE) tissues requires complex preprocessing, including washing steps, protein denaturing, or antigen retrieval, and finally enzymatic digestion of the tissues in liquids for several hours without major dislocation of the analyte biomolecules.

Enzyme digestion is usually carried out using set-ups developed by individual laboratories, like petri dishes [9,11] or so-called humidity boxes [8,12,13]. To ensure optimal and constant humidity and temperature for the enzyme on the tissue, saturated solutions of different salts are used [14], or boxes are placed in an oven or cell culture incubator. As this kind of procedure is difficult to standardize and requires substantial logistics and cross-site training for reproducible digestion results in multi-lab studies, it contributes to batch effects in MALDI-MSI and may limit the use of digestion methods in multi-site, industrial, or routine clinical environments [15,16]. Since enzymes only work in liquids, the wetness of the tissue and humidity of the closed digestion chamber are arguably the most critical factors for successful enzymatic digests. Unfortunately, high tissue wetness does not only result in effective enzymatic digestion, but often leads to dislocation of analytes [17]. This issue is more pronounced when heterogeneous tissue samples with different cell types are assessed to determine spatially resolved analytes in pharmaceutical research [2]. To prevent analyte dislocation and to guarantee highly standardized (reliable, controllable, reproducible) conditions, a fully software-controlled device, which enables monitoring and controlling of humidity in the chamber as well as of temperature directly under the sample slide and at the lid of the chamber was introduced. Through cyclic heating and cooling steps, this construction enables the controlled and repeated generation and disposition of microcondensate on the tissue. This consistent wetness of the tissue surface results in an optimized environment for enzymatic reactions while minimizing the spatial dislocation of analytes. Hence, the device presented here supports the lab-to-lab transfer of incubation protocols for any kind of on-tissue incubation. As an add-on in this device, all parts in contact with the reaction solution are made of anodized aluminum, which makes the device solvent-resistant and also potentially suitable for other applications (e.g., on-tissue chemical derivatization), where much harsher conditions are needed.

Currently, visualization of drugs and their potential metabolites in tissues represents an attractive application of MALDI-MSI as it provides essential spatial information to support pharmacokinetic/pharmacodynamic (PK/PD) modeling or optimizing drug absorption, distribution, metabolism, and excretion (ADME) properties [18,19]. *N*-Glycosylation, a common and complex post-translation modification (PTM), is of high analytical interest as it plays diverse roles that affect protein function both in normal physiology and disease conditions [20,21]. Thus, many of the commercially available therapeutic proteins are glycoproteins. For example, there is a great interest in increasing the therapeutic efficacy of protein drugs by manipulating the glycosylation patterns [22]. In addition, glycan signatures can be used for diagnostic and prognostic stratification of patients prior to pharmacotherapy [23,24,25]. However, the heterogeneous nature of the glycan profiles necessitates the development and application of robust protocols to better understand and analyze glycans of potential therapeutic importance. *N*-glycans were chosen for the application of this new device and for the development of an analytical workflow for fresh-frozen tissue that includes tissue preprocessing and optimized PNGaseF incubation conditions. The protocol was successfully exemplified for the evaluation of *N*-glycan distribution in mouse brain as well as for *N*-glycan-based k-Means segmentation of xenografted human U87 glioblastoma cells in CD1 nu/nu mouse brain based on MSI performed with an orthogonal MALDI-quadrupole-Time-of-Flight MS (MALDI-qToF-MS).

## 2. Results and Discussion

### 2.1. Digestion Chamber with Controlled Microcondensation on Tissue Slices

Spatially resolved analysis of larger or more complex biomolecules like tryptic peptides, *N*-glycans or endogenous enzymes requires consistent tissue digestions as part of MALDI-MSI workflows. Using this re-engineered device, the microcondensation on tissue slices was aimed to be controlled by having microdroplets for an efficient enzymatic digestion.

It was observed that the generated microcondensate on the slides was stable for only about 5–6 min. After this time, the microcondensate slowly dissolved. It was assumed that this evaporation was caused by the fact that the chamber was not hermetically sealed. As a consequence, the chamber was programmed to increase the base temperature to 39 °C every 5 min. This temperature increase helped to evaporate a little more water from the reservoirs and then induce microcondensation again by cooling to 37 °C. When testing the stability of this process over longer time periods (16 h) recommended in various protocols for on-tissue enzymatic digestion, similar extents of microcondensation were observed in the last cycle as in the first cycle (Figure S1).

To evaluate the wetness on top of the ITO slide under different condensation conditions, a water-sensitive paper was used to indicate the total wetness accumulated over an entire incubation period. Whenever the cover temperature (27 °C) was 10 °C lower compared to the base temperature (37 °C), no wetness could be detected on the slide even after 2 h of incubation. After reversing the temperature difference (cover 47 °C, base 37 °C) a bluish color could be observed on the water-sensitive paper. (Figure S2). This supported the finding from our design of experiments (DoE)-based method development that a difference of 10 °C between base and cover led to satisfying condensation on the slide. Furthermore, to distribute humidity homogenously inside the chamber, a 30 s ventilation step was introduced after the first 5 min initial phase and while heating up the base initially to 39 °C.

### 2.2. MALDI-MSI of N-Glycans in Fresh-Frozen Mouse Brain Using an Orthogonal qToF Mass Spectrometer

To test if this device can effectively be used for enzymatic on-tissue digests for MALDI-MSI, a workflow was adapted for *N*-glycan digestion of fresh-frozen tissue with PNGase F and MALDI-MSI was performed on an orthogonal MALDI timsToFflex mass spectrometer. The method development was done according to a factorial DoE approach, as published by Oetjen et al. [26] to test many aspects of multi-step experimental workflow (Figure S3). Based on the mean peak intensity and the occurrence of 59 predefined *N*-glycans masses [9] that served as quality criteria, we established the protocol reported in the Materials and Methods. Key parameters inside the digestion device include a digestion time of 4.5 h and removal of the microcondensate every 5 min by increasing the base temperature to 39 °C for 1 min.

**Figure 1:**
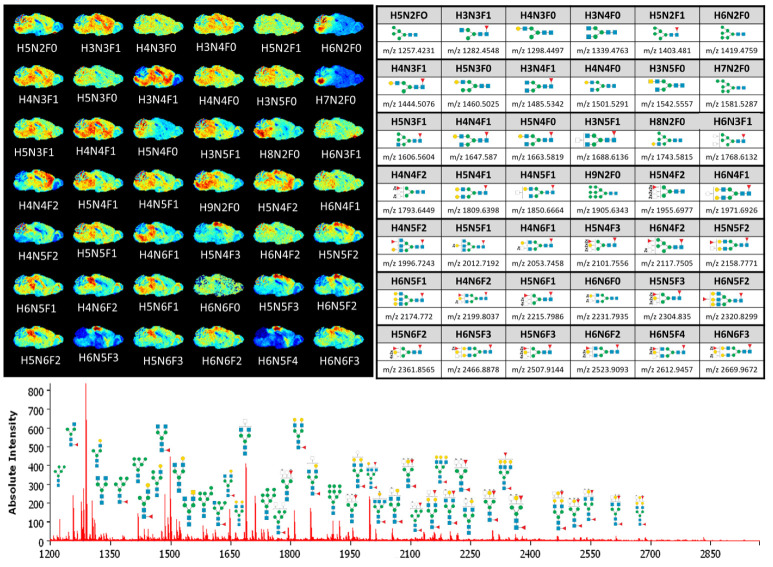
Application of the digestion device-based spatial *N*-glycan mapping workflow using timsToF flex MSI. Shown are 42 *m*/*z* distributions on fresh-frozen (FF) sagittal brain sections (**top left**) of the candidate corresponding *N*-glycans from a reference list (Toghi Eshgi et al.) [27] (**top right**). H indicates the number of hexoses, N the number of N-acetylhexosamines and F the number of fucoses in the carbohydrate moiety. The lower mass spectrum shows the absolute intensity distribution of the corresponding *m*/*z* values represented in the MS Image.

Afterwards fresh-frozen (FF) sagittal brain sections from CB57/6N mice (Figure 1 and Figure S4) were evaluated and the distribution of 42 *N*-glycans from the reference list of Toghi Esghi et al. [27] was considered. The clear differences in the spatial distribution of *N*-glycans, in particular, the clear distinction between white and gray matter in the cerebellum, confirmed the optimized conditions for microcondensate-based humidification without strong delocalization enabled by the digestion device. Although the distribution of 42 *N*-glycan *m*/*z* was described for FFPE tissue measured with DHB matrix [27], the distribution of *m*/*z* values representing oligomannose and non-fucosylated *N*-glycans in FF tissue looked similar. The reported distribution of the fucosylated *N*-glycans H4N4F2, H5N4F3, H5N5F2, and H6N5F4 in the cerebrum (isocortex and hippo-campal formation) in contrast to the *N*-glycan H4N6F2 was more localized in the brain stem (BS). However, we additionally observed that the oligomannose *N*-glycans that were described as being more abundant in the BS [27] were not distributed evenly throughout the whole BS but were predominantly present in the hindbrain and to a lesser extent in the cerebellum. In particular, H6N2F0, H7N2F0, H8N2F0, and H9N2F0 were characterized by their focused localization (Figure 1).

### 2.3. Differential Distribution of N-Glycans in Human Glioblastoma Cell-Derived Xenografts in Mice

**Figure 2:**
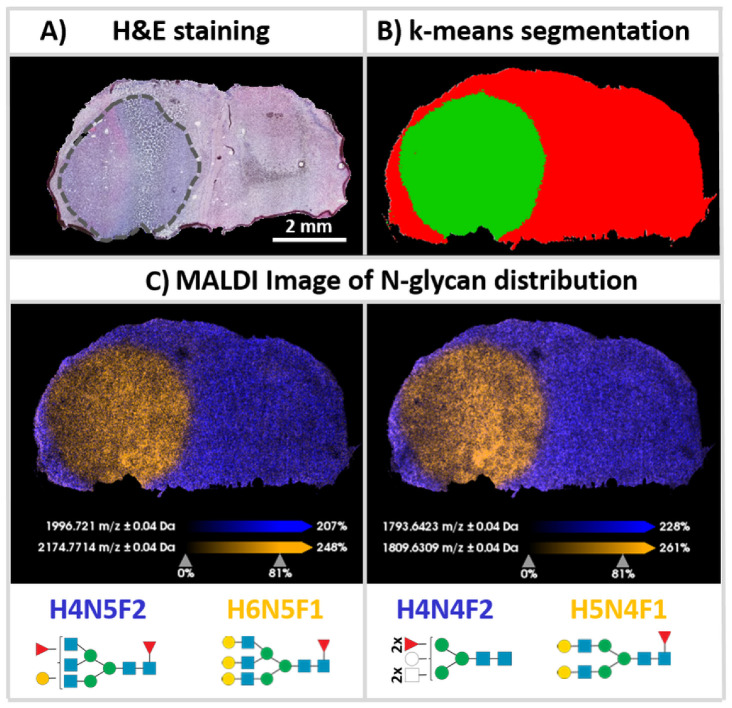
k-Means segmentation of mouse brains featuring tumors of human glioblastoma cell-derived xenografts. FF coronal sections of xenografted human U87 glioblastoma cells in CD1 nu/nu mouse brain were processed for *N*-glycan MSI. (**A**) H&E staining of an adjacent (and slightly distorted) tissue section. The black dotted line encircles the CDX tumor. (**B**) Result of k-means segmentation. (**C**) Orange segments indicate areas of xenograft tumor visualized by the *m*/*z* values of H6N5F1 [M + Na]^+^(*m*/*z* 2174.7714) (left) or H5N4F1 [M + Na]^+^ (*m*/*z* 1809.631) (right), whereas purple corresponds to the non-tumor area represented by the *m*/*z* value of H4N5F2 [M + Na]^+^ (*m*/*z* 1996.721) (left) or H4N4F2 [M + Na]^+^ (*m*/*z* 1793.642).

We had previously used tissue sections of mouse brains containing tumors of xenografted human U87 glioblastoma cells [28]. Here, we used analogous brains with cell line-derived xenografts (CDX) to investigate the degree of spatial dislocation that would correspond to the extensive mixing of the mouse brain and human CDX *N*-glycans at and around the xenograft border (Figure 2). Using the device and optimized protocol, a very sharp spatial separation between tumor and non-tumor tissue was achieved. Based on the previously used 42 *N*-glycan intervals established in k-means segmentation (Figure 2B), this led to a clear and spatially precise separation of both types of tissue when compared to the corresponding H&E staining of the adjacent (and slightly distorted) tissue section (Figure 2A). The precise separation of tumor and non-tumor in FF tissue supports the effective control of the digestion process. In addition, the signal intensities and mass errors of the *N*-glycans detected on the tissues (*n* = 3) were assessed to provide a preliminary perspective on the repeatability of the method. Out of 42 reference N-glycans [27], 32 *N*-glycans (76.20%) were commonly detected with a S/N ratio > 5 in all of the tissue sections with less than 50 ppm average mass errors between the theoretical and measured masses (Table 1).

Receiver-operating-characteristic (ROC) analysis of both segments (Figure 2B; tumor = green; non-tumor = red) resulted in five characteristic tumor-negative *N*-glycan structures, namely H3N4F1, H4N4F2, H5N2F0, H4N5F2, and H3N5F1. Similarly, high molecular specificity could be also observed in two replicates (Figures S5 and S6). Of these five *N*-glycans, three (H4N4F2, H4N5F2, and H3N5F1) have already been described as being downregulated in NS276 glioblastoma cell-derived xenografts [27].

In contrast, the ROC analysis also indicated five oligomannose *N*-glycans H5N4F1, H6N5F1, H5N4F0, H5N3F0, and H4N3F0, which are quite specific for tumor tissue of the human-derived xenografts. With the exception of H6N5F1, these *N*-glycans have also already been described as being upregulated in the NS276 glioblastoma xenografts model [27]. In our replicates (Figures S5 and S6) H5N4F1, H5N4F0, H5N3F0, and H4N3F0 were also among the top five tumor-specific *N*-glycan moieties. In one replicate (Figure S5), H6N5F1 was also ranked in the top five. These substantial parallels with glycosylation patterns in other glioblastoma models suggest that the inherent diagnostic value of *N*-glycosylation patterns should prompt more extensive clinical research. This outlook underscores the need for *N*-glycan MSI with a high degree of standardization using a specialized digestion device.

## 3. Materials and Methods

### 3.1. Materials

All solvents were obtained from VWR (Bruchsal, Germany) and Sigma-Aldrich Chemie GmbH (Taufkirchen, Germany) in the highest available purity. The MALDI matrix α-cyano-4-hydroxycinnamic acid (HCCA) and indium tin oxide- (ITO-) coated conductive glass slides were obtained from Bruker Daltonik GmbH (Bremen, Germany). Recombinant endoglycosidase PNGase F (Cat. No. 36404.01) was purchased from SERVA Electrophoresis GmbH (Heidelberg, Germany). n-Octyl-β-d-glucopyranoside was purchased from Sigma-Aldrich Chemie GmbH (Taufkirchen, Germany).

### 3.2. Animal Model and Tissue Samples

Porcine brain for method development was obtained from a local slaughterhouse and stored at −80 °C until usage. Fresh-frozen brains from 12 week-old female wild-type (wt) C57BL/6N mice were obtained from the Karlsruhe Institute of Technology (KIT). Animal studies on engrafted CD1 nu/nu mice were conducted at the German Cancer Research Center (DKFZ, Heidelberg, Germany). The implementation of the animal studies was based on the National Institute of Health guidelines “Guide for the Care and Use of Laboratory Animals” under supervision by institutional animal protection officials. The animal experiments were approved by government authorities (Regierungspräsidium Karlsruhe, Germany, approval number: G-179/17).

Frozen tissue samples were sectioned at 10 μm thickness using a Leica CM1950 cryostat (Leica Biosystems, Nussloch, Germany) at −15 °C chamber temperature and thaw-mounted onto indium tin oxide (ITO) coated glass slides (Bruker Daltonik, Bremen, Germany). Slides were then dried in a vacuum desiccator for 15 min at RT.

### 3.3. Re-Engineered Digestion Chamber with Controlled Microcondensation

**Figure 3:**
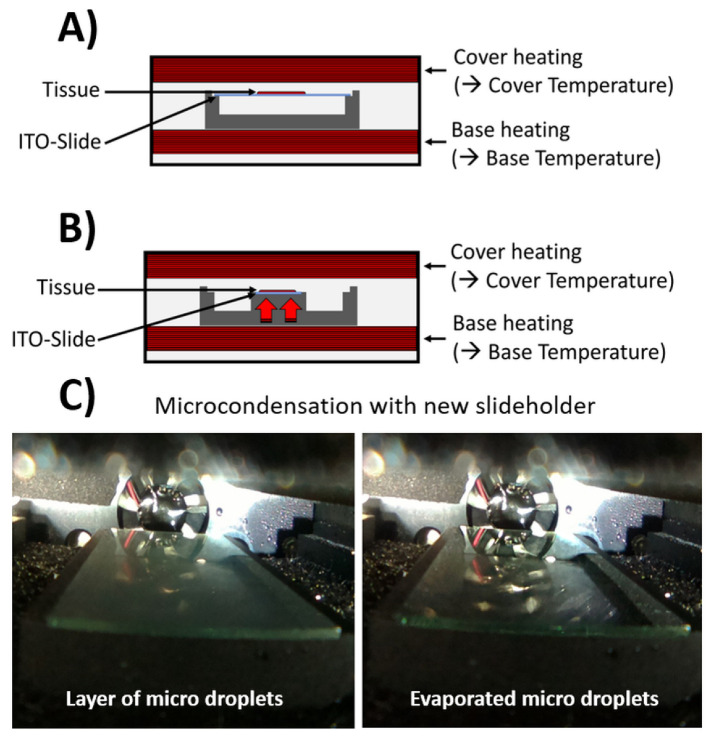
Design of the digestion chamber for controlled condensation of microdroplets on tissue slices on ITO slides. In contrast to an (**A**) old design of a digestion chamber, (**B**) a heated slide holder was constructed for this computer-controlled device that allows for direct and fast regulation of the base temperature of the ITO-slide. (**C**) Periodic temperature cycling results in slides covered with a thin, homogenous layer of condensed microdroplets (left panel), which evaporate when the base temperature is increased again (right panel).

To develop more standardized workflows with a robust, computer-controlled device, an apparatus that enabled controlled microcondensation on tissue slices was engineered. An incubation chamber for on-tissue enzyme digests based on a commercially available device, SunDigest Incubator (SunChrom, Friedrichsdorf, Germany), was re-engineered [29] (Figure 3A). The device presented here enabled software-controlled cyclic temperature control (4–50 °C) between the heated lid and the heated slide holder within the chamber (Figure 3B and Figure S7). This slide holder allowed for direct heat transfer to the ITO-slide for fast and controlled changes in temperature. Furthermore, the slide holder inside the digestion device was designed in a way to introduce a heat difference between the slide (“base temperature”) and the surrounding air, which is mainly controlled by the heated lid (“cover temperature”).

Warm air has a higher maximum possible vapor pressure and can thus absorb more water than cold air, which results in more condensation when deposited on cooler surfaces. Therefore, the lid of the incubation chamber is actively temperature-regulated to prevent any water condensation, which could cause water to drip onto the tissue and ruin the sample. The temperature difference between ITO-slide (37 °C) and the inner chamber environment, which is regulated through the cover temperature (47 °C), leads to a fine layer of microdroplets on top of the ITO-slide (Figure 3C). In a Supplementary Video, the microcondensation inside the chamber during the incubation run is shown. These microdroplets ensure controlled wetness on the tissue and maintain the process of on-tissue enzymatic digestion between the states of too dry, i.e., not promoting enzyme function, and too wet, i.e., promoting analyte dislocation.

### 3.4. Tissue Processing

The best results were obtained with the following workflow and parameters on fresh-frozen tissue:

1. Detergent treatment for denaturation: Denaturation of glycoproteins prior to enzymatic digestion has been shown to increase the deglycosylation efficiency [30]. Twenty µL detergent solution (20 mg n-octyl-β-d-glucopyranoside in 7 µL 2-mercaptoethanol and 993 µL ddH_2_O) was applied onto each tissue slice and left for 10 min at RT. The detergent solution was removed directly in step 2 by dipping the tissue slices mounted onto the glass sides in organic solvents.
2. Tissue washing and delipidation: For removal of lipid background and detergent solution, a six-step washing protocol was used: first, 100% ethanol (EtOH) for 1 min; second and third, Carnoy’s fluid (60% EtOH/30% chloroform/10% acetic acid *v*/*v*/*v*) for 2 min; fourth, 100% EtOH for 1 min; fifth and sixth, 70% EtOH for 2 min; seventh and eighth, ddH_2_O for 2 min. After washing the tissue slices, the glass slides were dried for 5 min under vacuum at RT.
3. Application of PNGase F: To cleave *N*-glycans from proteins, 150 µL PNGase F solution (100 ng/µL PNGase in ddH_2_O) was applied to each slide using the SunCollect MALDI Sprayer equipped with a syringe pump (SunChrom, Friedrichsdorf, Germany) in 10 layers using a line distance of 1 mm at RT. Further parameters regarding enzyme application were: distance between tissue and spray head was z = 30 mm, flow rate 10 µL/min, speed 1080 mm/min (“medium 4”) and air pressure at 2.5 bar.
4. Enzymatic digestion using the device: Both cotton sponges from the device were dipped into ddH_2_O until they were totally wet and placed in the instrument. The glass slides with the tissue sample up were placed on top of the heating bar and incubated for 4.5 h using an iterative temperature program (base 37–39 °C; cover 47 °C).
5. Matrix application: A-cyano-4-hydroxycinnamic acid (HCCA, 7 mg/mL in 50% acetonitrile + 0.1% trifluoroacetic acid) was deposited in 15 layers using a line distance of 2 mm at RT using the SunCollect MALDI Sprayer equipped with a dispenser system (SunChrom). Further parameters regarding matrix application were: z = 30 mm, flow rate 10 µL/min (first layer) and 15 µL/min (last layers), speed 900 mm/min and air pressure at 2.5 bar.

### 3.5. MALDI-qToF MS Imaging

Data acquisition was performed on a timsToF fleX (Bruker Daltonics, Bremen, Germany) in positive ion mode in the range of *m*/*z* 900–3200.

Spectra were recorded using 600 shots per pixel with a laser repetition rate of 10 kHz using a 50 × 50 µm step size. Transfer parameters were as follows: Funnel 1 RF 500 Vpp; Funnel 2 RF 500 Vpp; Multipole RF 500 Vpp; Deflection Delta 70 V; MALDI Plate Offset: 50 V. Quadrupole parameters: Ion energy 5.0 eV; Low Mass *m*/*z* 900. Focus PreToF Parameters: Transfer Time 145 µs; PrePulse Storage 28 µs.

External calibration was done in the electrospray mode using ESI-Low Concentration Tuning Mix (Agilent Technologies, Santa Clara, CA, USA)

### 3.6. Image Reconstruction and Data Analysis

All images were reconstructed and processed using SCiLS Lab MVS 2020a Pro (Bruker Daltonics). Datasets measured on the MALDI-qToF system were Root Mean Square-normalized. Bisecting K-Means segmentation was performed based on our reference list of 42 *N*-glycans [27] without any denoising, using a correlation distance metric on every individual spectrum. ROC analysis to find discriminating values was performed on all individual spectra based on the reference list described before.

### 3.7. Hematoxylin and Eosin (H&E) Tissue Staining

For hematoxylin and eosin staining, adjacent sections were mounted onto HistoBond^®^+M (Paul Marienfeld GmbH & Co. KG, Lauda-Königshofen, Germany) adhesive microscope slides. The sections were immersed into Mayer’s hematoxylin solution (2 min), immersed in tap water (3 min) and dipped in distilled water afterwards. To enhance the nuclei staining, slides were immersed into 0.3% acid alcohol (0.3% acid alcohol in 70% ethanol solution, 30 s), dipped in distilled water, immersed into bluing solution (10 g NaHCO_3_ + 100 g MgSO_4_ in 5 L H_2_O) for 2 min and then dipped in distilled water. Then, the sections were immersed in eosin solution for 2 min and rinsed with distilled water. The slices were each dehydrated for 2 min in various alcohol solutions (80%, 96%, 100% ethanol) and cleared with xylol solution. Slides were covered with Eukitt mounting medium (VWR, Bruchsal, Germany) and coverslips. Pictures of H&E-stained histological sections were obtained with an Aperio CS2 Scanner (Leica Biosystems, Nussloch, Germany) at 20× magnification.

## 4. Conclusions

On-tissue digestion is one of the most critical factors contributing to the molecular information and spatial resolution of the resulting ion images. A new device for on-tissue enzymatic reactions was designed and constructed that enables controlled enzyme digestions in MSI applications. The possibility of software-based monitoring and tight control of the desired reaction environment ensures a standardizable and effective process. The new construction of the slide holder allows direct heat transfer to the sample slide. With this, the process of cyclic heating controls the regular, cyclic, and effective formation of a fine layer of microdroplets on the tissue by microcondensation. This, in turn, provides a rather stable and spatially confined wetness, and commonly observed analyte delocalization can be attenuated. Using benchmarks for identification of *N*-glycans, potential diagnostic, prognostic and pharmacodynamic biomarkers for patient stratification and target engagement, in mouse brain, and for spatial confinement of the digest, we exemplified the utility of this device. As a future perspective, the use of this device may not only be limited to other enzymatic approaches (e.g., protease digest), but due to its solvent-resistant construction with anodized aluminum, it may also be useful in chemical reactions on-tissue such as derivatizations in a controlled environment. With the possibility of deeper control of the reaction conditions on the tissue section using the new device, a cornerstone has been laid for the application of enzymatic digestion or chemical derivatization not only in research but also in the clinical and industrial environment.

## Figures and Tables

**Table 1:** Common *N*-glycans detected in human glioblastoma cell-derived xenografts (*n* = 3).

*N*-glycan Species	Theoretical *m*/*z*	Tissue #1			Tissue #2			Tissue #3		
		*m*/*z*	Relative Intensity	Mass Error (ppm)	*m*/*z*	Relative Intensity	Mass Error (ppm)	*m*/*z*	Relative Intensity	Mass Error (ppm)
H6N2F0	1419.476	1419.474	3.68	1.4	1419.476	4.09	0.2	1419.460	2.93	11.1
H4N3F1	1444.508	1444.543	0.51	24.8	1444.502	0.64	3.7	1444.477	0.35	21.1
H3N4F1	1485.534	1485.553	4.09	12.6	1485.532	6.34	1.3	1485.463	3.61	47.8
H3N5F0	1542.556	1542.576	0.63	12.9	1542.566	0.58	6.4	1542.462	0.40	60.9
H7N2F0	1581.529	1581.534	2.03	3.6	1581.504	2.43	15.8	1581.514	1.51	9.5
H5N3F1	1606.560	1606.579	0.52	11.7	1606.554	0.62	3.8	1606.547	0.34	8.1
H4N4F1	1647.587	1647.564	1.11	13.9	1647.584	1.46	1.9	1647.498	0.87	54
H5N4F0	1663.582	1663.577	0.73	2.8	1663.611	0.62	17.8	1663.489	0.47	56.1
H3N5F1	1688.614	1688.622	5.82	4.9	1688.613	7.04	0.2	1688.517	5.03	57.1
H8N2F0	1743.582	1743.569	1.68	7	1743.578	2.19	1.8	1743.504	1.31	44.4
H4N4F2	1793.645	1793.642	1.11	1.6	1793.657	1.54	6.8	1793.660	0.77	8.1
H5N4F1	1809.640	1809.631	1.16	4.9	1809.651	1.39	6.0	1809.556	0.82	8.7
H4N5F1	1850.666	1850.656	1.20	5.6	1850.662	1.41	2.1	1850.631	0.86	18.9
H9N2F0	1905.634	1905.603	1.74	16.5	1905.627	2.18	3.8	1905.632	1.45	1.1
H5N4F2	1955.698	1955.667	0.64	15.5	1955.703	0.74	2.6	1955.741	0.41	22.1
H6N4F1	1971.693	1971.705	0.28	6.1	1971.681	0.25	5.7	1971.731	0.16	19.5
H4N5F2	1996.724	1996.721	2.31	1.7	1996.735	2.91	5.7	1996.741	1.78	8.5
H5N5F1	2012.719	2012.713	0.38	3	2012.71	0.40	4.7	2012.807	0.22	43.7
H4N6F1	2053.746	2053.746	0.53	0.1	2053.738	0.57	3.8	2053.826	0.36	39.3
H5N4F3	2101.756	2101.76	0.17	2	2101.746	0.21	4.5	2101.875	0.09	56.6
H6N4F2	2117.751	2117.724	0.17	12.5	2117.749	0.15	0.8	2117.863	0.09	52.9
H5N5F2	2158.777	2158.805	0.37	13.1	2158.752	0.46	11.4	2158.824	0.22	21.7
H6N5F1	2174.772	2174.771	0.16	0.3	2174.780	0.19	3.6	2174.792	0.08	9.1
H4N6F2	2199.804	2199.789	0.49	6.7	2199.805	0.53	0.4	2199.852	0.33	21.8
H5N6F1	2215.799	2215.826	0.19	12.4	2215.783	0.21	7.1	2215.931	0.12	59.9
H5N5F3	2304.835	2304.872	0.51	16	2304.818	0.64	7.3	2304.954	0.31	51.5
H6N5F2	2320.830	2320.86	0.14	13	2320.845	0.17	6.4	2320.974	0.08	61.9
H5N6F2	2361.857	2361.844	0.29	5.5	2361.872	0.32	6.7	2361.944	0.18	37
H6N5F3	2466.888	2466.852	0.19	14.4	2466.908	0.26	8.4	2466.913	0.10	10.3
H5N6F3	2507.914	2507.933	0.16	7.5	2507.936	0.19	8.4	2507.868	0.11	18.6
H6N6F2	2523.909	2523.921	0.08	4.7	2523.913	0.09	1.6	2523.845	0.05	25.4
H6N5F4	2612.946	2612.99	0.19	17	2612.947	0.25	0.3	2612.960	0.11	5.4

*N*-glycans only detected in three of the tissues with S/N > 5 were selected.

## Data Availability

Data is contained within the article or Supplementary Material.

## Supplementary Materials

The following supporting information can be downloaded at: https://www.mdpi.com/article/10.3390/ph15111356/s1. Figure S1: Time-dependent stability of on-slide micro-condensation; Figure S2: Testing the wetness on the ITO-Slide during incubation using water sensitive paper; Figure S3: Schematic overview of workflow for tissue pre-measurement processing; Figure S4: H&E staining of an adjacent sagittal section of a wild-type C57BL/6N mouse brain; Figure S5: Replicate I: K-Means segmentation of xenografted human U87 glioblastoma cells in CD1 nu/nu mouse brain; Figure S6: Replicate II: K-Means segmentation of xenografted human U87 glioblastoma cells in CD1 nu/nu mouse brain; Figure S7: Design of digestion device for controlled temperature and humidity.
