# Supplementary material for: Device-Controlled Microcondensation for Spatially Confined On-Tissue Digests in MALDI Imaging of N-Glycans
Source: Pharmaceuticals (Basel). 2022 Nov 3;15(11):1356. doi: 10.3390/ph15111356 (PMC9698097; doi:10.3390/ph15111356)
Supplement: Supplementary file 1 [file pharmaceuticals-15-01356-s001.zip › pharmaceuticals-1937522-supplementary.pdf]

Supporting Information

Device-controlled microcondensation for spatially confined on-tissue digests in MALDI imaging of N-glycans

Annabelle Fülöp[1,#], Christian Marsching[1,#], Frederik Barka[2,#], Yasemin Furtun Ucal[1], Pauline Pfänder[3,4], Christiane A. Opitz[3,5], Günes Barka[2], Carsten Hopf[1,6,7*]

1 Center for Mass Spectrometry and Optical Spectroscopy (CeMOS), Mannheim University of Applied Sciences, Mannheim, Germany
2 SunChrom Wissenschaftliche Geräte GmbH, Friedrichsdorf, Germany
3 Metabolic Crosstalk in Cancer, German Consortium of Translational Cancer Research (DKTK), German Cancer Research Center (DKFZ), Heidelberg, Germany
4 Faculty of Bioscience, Heidelberg University, 69120 Heidelberg, Germany
5 Neurology Clinic and National Center for Tumor Diseases, Heidelberg, Germany
6 Medical Faculty, Heidelberg University, Heidelberg, Germany
7 Mannheim Center for Translational Neuroscience (MCTN), Mannheim, Germany
# equal contribution

[*] Corresponding Author: Prof. Dr. Carsten Hopf, Email: c.hopf@hs-mannheim.de

SI Figure 1 (Figure S1)	Time-dependent stability of on-slide micro-condensation	S-2
SI Figure 2 (Figure S2)	Testing the wetness on the ITO-Slide during incubation using water sensitive paper	S-3
SI Figure 3 (Figure S3)	Schematic overview of workflow for tissue pre-measurement processing	S-3
SI Figure 4 (Figure S4)	H&E staining of an adjacent sagittal section of a wild-type C57BL/6N mouse brain	S-4
SI Figure 5 (Figure S5)	Replicate I: K-Means segmentation of xenografted human U87 glioblastoma cells in CD1 nu/nu mouse brain	S-4
SI Figure 6 (Figure S6)	Replicate II: K-Means segmentation of xenografted human U87 glioblastoma cells in CD1 nu/nu mouse brain	S-5
SI Figure 7 (Figure S7)	Design of digestion device for controlled temperature and humidity.	S-5

SI Supplementary Figures

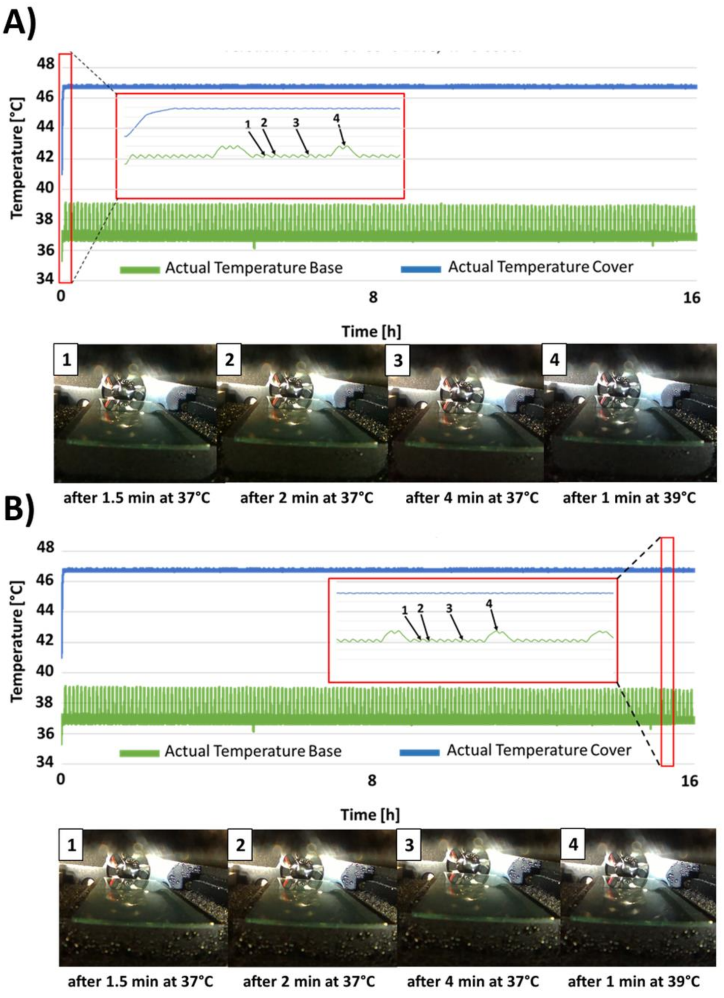

Figure S1: Time-dependent stability of on-slide microcondensation. (a) No difference could be seen in the resulting microcondensation evaluating the droplet layer (1-3) and the water on the slide was evaporated (4) over a time span of 15 h 30 min. **(b)** In the microcondensation step after 15 h 30 min (b), each monitored time point in a cycle gave the same results as observed in the first one starting after the initial phase of 5 min (a).

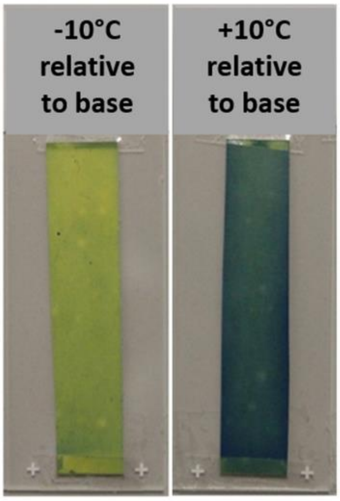

Figure S2: Testing of wetness on the ITO-Slide during incubation using water-sensitive paper. A temperature difference of +10°C between the base and cover of the device leads to a satisfying result. In this case (right) the strong bluish coloring indicates a higher level of condensation on the ITO-slide, compared to a temperature difference of -10°C and missing condensation on the paper (left).

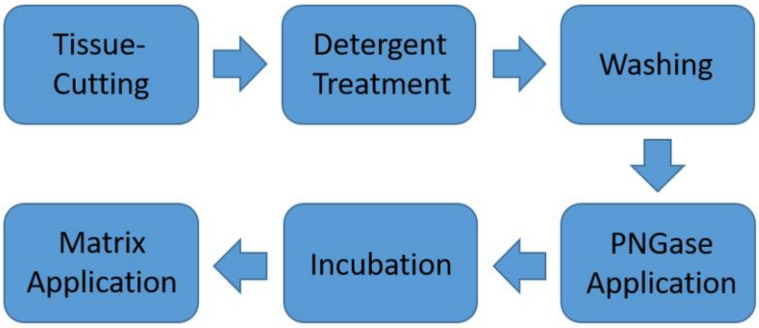

Figure S3: Scheme of the tissue-processing workflow. The workflow required before the analysis of N-glycan moieties with MALDI-MSI. Details of the individual steps are described in the method section.

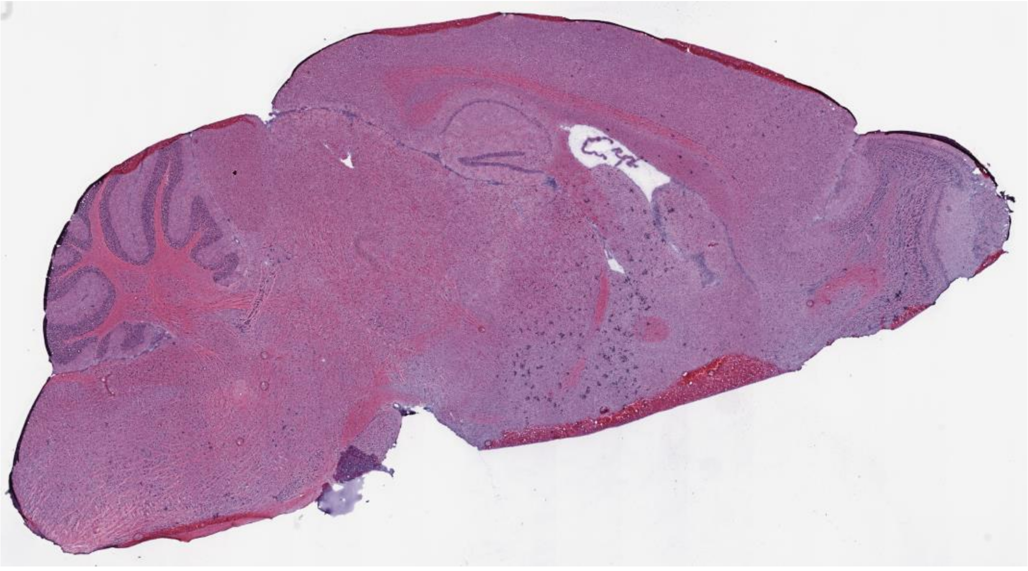

Figure S4: H&E staining of an adjacent sagittal section (to the sections used to map the N-glycan spatial distribution (Fig. 1)) of a wild-type C57BL/6N mouse brain.

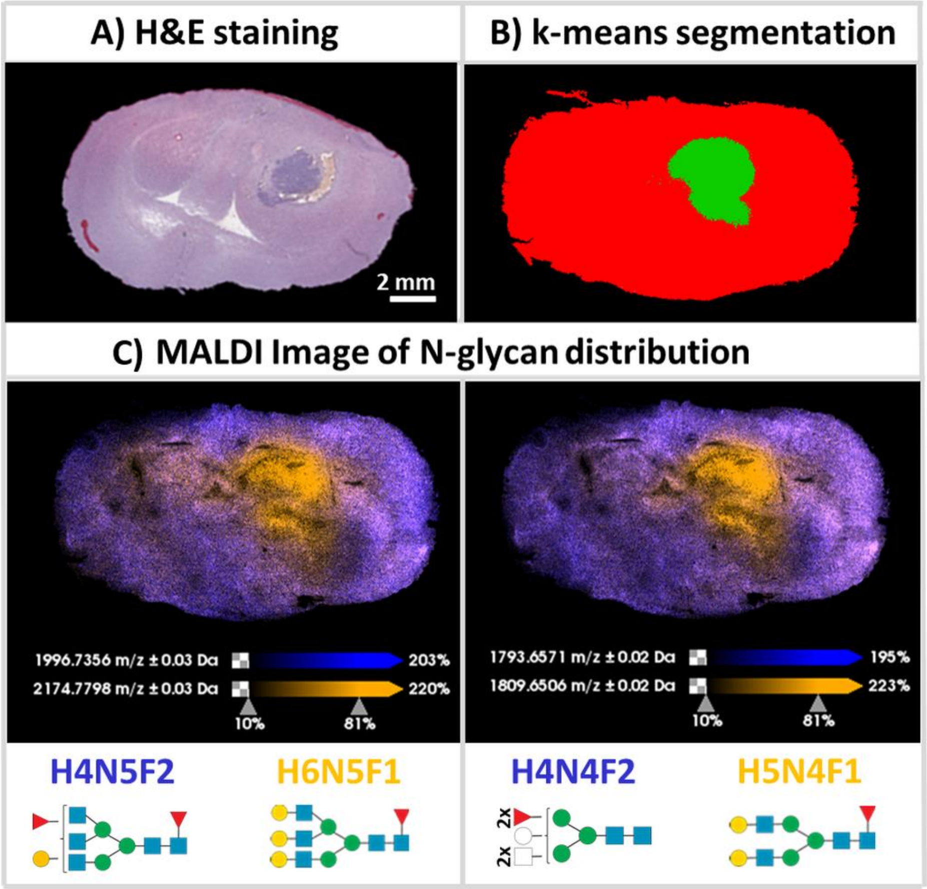

Figure S5: First replicate of the measurement used for Fig. 2. (a) H&E staining of an adjacent tissue section. (b) Result of k-means segmentation. (c) Orange segments indicate areas of xenograft tumor visualized by the m/z values of H5N4F1 $[M+Na]^+$ (m/z 1809.6506) or H6N5F1 $[M+Na]^+$ (m/z 2174.7798), whereas purple corresponds to the non-tumor area represented by the m/z value of H4N4F2 $[M+Na]^+$ (m/z 1793.6571) or H4N5F2 $[M+Na]^+$ (m/z 1996.7356).

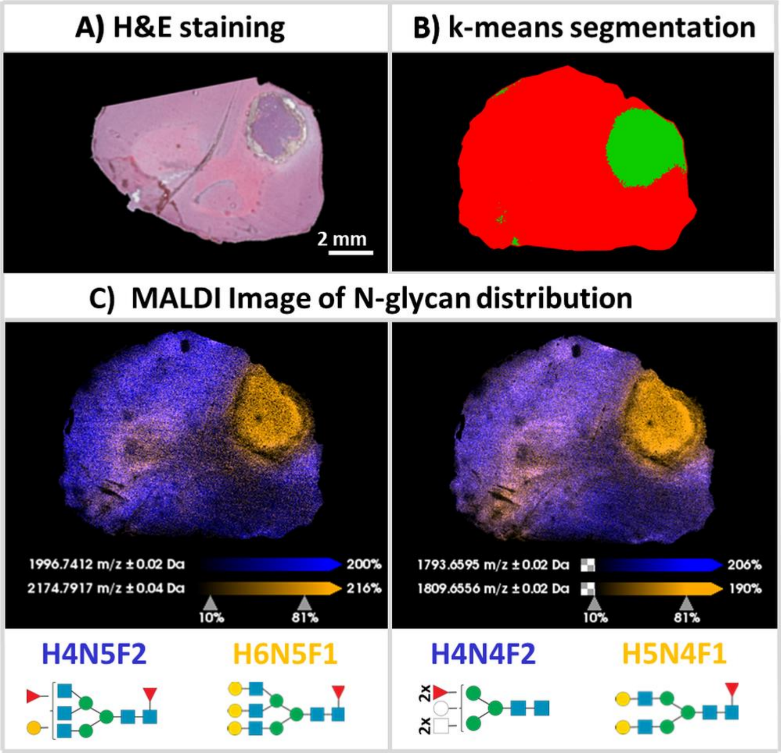

Figure S6: Second replicate of the measurement used for Fig. 2. (a) H&E staining of an adjacent (and slightly distorted) tissue section. (b) Result of k-means segmentation. (c) Orange segments indicate areas of xenograft tumor visualized by the m/z values of H5N4F1 $[M+Na]^+$ (m/z 1809.6556) or H6N5F1 $[M+Na]^+$ (m/z 2174.7917), whereas purple corresponds to the non-tumor area represented by the m/z value of H4N4F2 $[M+Na]^+$ (m/z 1793.6595) or H4N5F2 $[M+Na]^+$ (m/z 1996.7412).

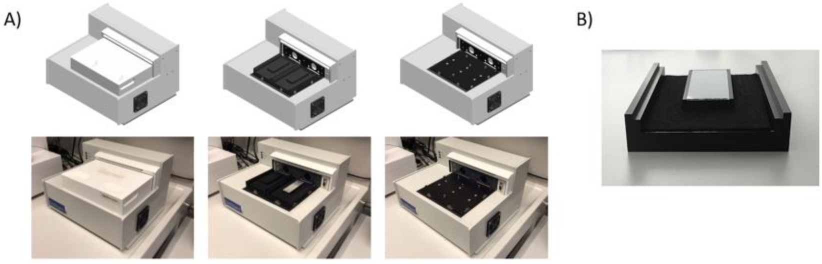

Figure S7: SunDigest 2.0. (a) Schematic drawing of the temperature- and humidity-controlled on-tissue digestion device and the demonstrator used in the laboratory. **(b)** The device contains a slide holder made of anodized aluminum.
